# Effective Mechanisms for Improving Seed Oil Production in Pennycress (*Thlaspi arvense* L.) Highlighted by Integration of Comparative Metabolomics and Transcriptomics

**DOI:** 10.3389/fpls.2022.943585

**Published:** 2022-07-14

**Authors:** Christopher Johnston, Leidy Tatiana García Navarrete, Emmanuel Ortiz, Trevor B. Romsdahl, Athanas Guzha, Kent D. Chapman, Erich Grotewold, Ana Paula Alonso

**Affiliations:** ^1^Department of Biological Sciences, BioDiscovery Institute, University of North Texas, Denton, TX, United States; ^2^Department of Biochemistry and Molecular Biology, Michigan State University, East Lansing, MI, United States

**Keywords:** ascorbate, biofuel, cell wall, central carbon metabolism, erucic acid (C22:1), lipid droplet, *Thlaspi arvense*, threonine aldolase

## Abstract

Pennycress is a potentially lucrative biofuel crop due to its high content of long-chain unsaturated fatty acids, and because it uses non-conventional pathways to achieve efficient oil production. However, metabolic engineering is required to improve pennycress oilseed content and make it an economically viable source of aviation fuel. Research is warranted to determine if further upregulation of these non-conventional pathways could improve oil production within the species even more, which would indicate these processes serve as promising metabolic engineering targets and could provide the improvement necessary for economic feasibility of this crop. To test this hypothesis, we performed a comparative biomass, metabolomic, and transcriptomic analyses between a high oil accession (HO) and low oil accession (LO) of pennycress to assess potential factors required to optimize oil content. An evident reduction in glycolysis intermediates, improved oxidative pentose phosphate pathway activity, malate accumulation in the tricarboxylic acid cycle, and an anaplerotic pathway upregulation were noted in the HO genotype. Additionally, higher levels of threonine aldolase transcripts imply a pyruvate bypass mechanism for acetyl-CoA production. Nucleotide sugar and ascorbate accumulation also were evident in HO, suggesting differential fate of associated carbon between the two genotypes. An altered transcriptome related to lipid droplet (LD) biosynthesis and stability suggests a contribution to a more tightly-packed LD arrangement in HO cotyledons. In addition to the importance of central carbon metabolism augmentation, alternative routes of carbon entry into fatty acid synthesis and modification, as well as transcriptionally modified changes in LD regulation, are key aspects of metabolism and storage associated with economically favorable phenotypes of the species.

## Introduction

The need for renewable fuel sources has been greatly highlighted in recent years by the growing world population and increasing concern over environmental impacts of conventional fossil fuel consumption (Thelen and Ohlrogge, 2002; Banković-Ilić et al., 2012). Oilseed crops have gained prominence in the search for biofuel sources due to recent improvements in the processing of their constituent oils (Kumar et al., 2016). The global oleochemical industry was valued at USD 25.8 billion in 2020, and is expected to display an annual growth rate of 6.5% from 2021 to 2027 (Astute Analytica, 2021). By 2025, the United States’ demand for renewable oils is expected to increase to a value of over USD 3 billion (Grand View Research, 2017). Additionally, energy security has been cited by both transportation and military sectors as a major incentive for producing renewable fuel sources that reduce the unpredictability associated with relying on imported fossil fuels (Drenth et al., 2014).

Native to Eurasia, pennycress (*Thlaspi arvense* L.) is a cool-season annual plant species well-adapted to North American lands, even those of marginal quality (Sedbrook et al., 2014; Zanetti et al., 2019). Natural pennycress accessions accumulate up to 36% of seed oil on a per weight basis, with production predominantly taking place in developing embryos (Moser et al., 2009b). Of particular interest is the fatty acid (FA) composition of the oil, approximately 33% of which is erucic acid, a 22-carbon monounsaturated FA (Moser et al., 2009b; Sedbrook et al., 2014). The high content of erucic acid in pennycress seed oil, along with other minor unsaturated FAs, contributes to its favorable profile for aviation fuel, including optimal combustion speed and desirable low temperature flow properties (Moser et al., 2009a). In addition to the chemical properties of its seed oil, the agronomic practices associated with successful pennycress cultivation make it interesting to growers and the agricultural sector. Government agencies have stressed that renewable fuels must be sourced from a non-food crop to minimize the impact on domestic food supplies (Blakeley, 2012). Pennycress has been identified as a lucrative candidate for introduction into crop rotations with major commercial crops like corn and soybean, because aerial broadcast of seed in the fall as a winter cover crop allows for combine harvesting the following spring (Dose et al., 2017; Markel et al., 2018; Mousavi-Avval and Shah, 2020). Not only does this benefit growers as an additional source of income, but also provides canopy cover that suppresses weed pressure, provides ecosystem services for pollinators, and maintains the soil coverage that provides erosion control and minimizes nutrient runoff (Dorn et al., 2015; Eberle et al., 2015; Johnson et al., 2015).

Despite the plethora of potential uses of pennycress seed oil, the existing germplasm must be improved *via* metabolic engineering and breeding to ensure profitability. A wide variety of oil content has been observed across natural accessions of pennycress, and relatively few, if any, of these accessions provide optimal seed oil yields as is (Cocuron et al., 2018; Cubins et al., 2019). While knowledge on FA and lipid metabolism in plants as a whole is strong, specific metabolic characteristics of pennycress require further elucidation for the improvement of its oil-related traits.

Seeds of the Brassicaceae family are known to generate carbon precursors for plastidic *de novo* FA biosynthesis *via* glycolytic flow and reductant *via* the oxidative pentose phosphate pathway (OPPP) activity (Dennis and Miernyk, 1982; Schwender et al., 2003; Baud and Lepiniec, 2009). Metabolic models, including those in pennycress, show strong evidence that much of this occurs *via* the conversion of phosphoenolpyruvate (PEP) and/or malate into pyruvate, which is then further broken down into acetyl-CoA, the main carbon building block for *de novo* FA synthesis (Kubis et al., 2004; Cocuron et al., 2014; Tsogtbaatar et al., 2020). The *de novo* synthesis of FAs in the plastid also requires ATP and reductant (NADPH/NADH), which along with carbon precursors can be generated *via* photosynthesis in green heterotrophic seeds (Hills, 2004; Baud and Lepiniec, 2010; Tsogtbaatar et al., 2015). The first desaturation of fatty acids takes place in the plastid, namely *via* stearoyl-ACP Δ9 desaturase, which is required for monounsaturated fatty acid production, including erucic acid (Subedi et al., 2020).

FAs produced in the plastid are released into the cytoplasm to produce an acyl-CoA pool, which contains FAs available for further modification and oil synthesis (Cagliari et al., 2011). In addition to the OPPP, the activity of the tricarboxylic acid (TCA) cycle is heavily involved in the production of carbon and/or reductant for extra-plastidic FA elongation in pennycress, which is required for the high production of erucic acid along with other long-chain and unsaturated FAs (Moser et al., 2009a; Claver et al., 2017; Tsogtbaatar et al., 2020). Elongation of FAs in the acyl-CoA pool takes place through a multi-enzyme complex, including an ER-localized condensing enzyme, 3-ketoacyl-CoA synthase (KCS; Millar and Kunst, 1997; Joubès et al., 2008). Incorporation of FAs into storage lipids then occurs *via* the Kennedy pathway, which involves incorporation into glycerol backbones to form triacylglycerols (TAGs; Kennedy, 1961; Romsdahl et al., 2021). Additional acyl-CoA-independent pathways can incorporate acyl chains directly to form TAGs as well (Dahlqvist et al., 2000; Lu et al., 2009; Cagliari et al., 2011; Hu et al., 2012; Wang et al., 2012; Kim and Chen, 2015).

The power of comparative “omics” approaches to study the intraspecific variation in oilseeds has been displayed in high and low oil maize accessions, where both increased incorporation of carbon (as glucose 6-phosphate) into the plastid and action of the plastidic malic enzyme were noted to result in higher oil accumulation and thus serve as potential targets for engineering (Cocuron et al., 2019). Furthermore, detailed metabolomic and ^13^C-labeling analyses have identified several non-conventional mechanisms (i.e., unusual and contrary to classical reports in the literature) involved in efficient oil production in pennycress (Tsogtbaatar et al., 2015, 2020). These include reversable isocitrate dehydrogenase activity, thus fixing CO_2_ to sustain FA elongation *via* citrate, and the incorporation of plastidic CO_2_ *via* Rubisco that was released by other metabolic reactions (Tsogtbaatar et al., 2020).

While these non-conventional mechanisms are associated with higher efficiency of oil production in pennycress compared to other species, it is to be determined if their upregulation can further improve oil production in pennycress itself. The comparative approach has promise in pennycress, as representative high and low oil accessions are available with differing biomass compositions. The purpose of this research was to perform a comprehensive comparative analysis of high and low-oil pennycress accessions *via* metabolomics and transcriptomics in order to identify which components of metabolism are likely key to boosting the production of the desirable oil, from *de novo* FA synthesis all the way to oil storage. The results indicated that mechanisms powering *de novo* FA synthesis associated with higher oil production cover early central carbon metabolism all the way to organization and stability of end-product lipid droplets. These mechanisms serve as valuable targets for improving oil production in pennycress.

## Materials and Methods

### Plant Materials

A representative accession producing a high percent weight per weight (% w/w) oil content (HO) at maturity (USDA-GRIN Acc. ‘Ames 32872’) and another accession containing low % w/w oil content (LO; USDA-GRIN Acc. ‘Ames 31500’) were chosen from existing seed stocks of USDA-GRIN pennycress accessions (Supplementary Figure S1). Seed stocks were stored at 4°C. Plant growth was carried out in accordance with Tsogtbaatar et al. (2015). Seeds of HO were immediately planted in soil and required no pretreatment for adequate germination. Pedicels of newly-opened flowers were hand-pollinated and tagged daily with acrylic paint to keep record of the age of developing siliques for embryo collection.

### Embryo Collection

For biomass and metabolomics analysis, siliques were selected according to how many days after pollination (DAP) had elapsed in accordance with Tsogtbaatar et al. (2015) and Cocuron and Alonso (2014). For embryos used in transcriptomics analysis, excised embryos were placed into an autoclaved microcentrifuge tube containing liquid nitrogen. Following collection of these embryos, microcentrifuge tubes were immediately transferred to a −80°C freezer prior to being capped following evaporation of all remaining liquid nitrogen in the tube. Embryos used for transcriptomics analysis were collected using autoclaved forceps and a microscope treated with RNase Zap (MilliporeSigma, Burlington, MA). For all analyses, the earliest stage selected for LO was 11 DAP and the earliest stage selected for HO was 10 DAP; these stages were in consistency with the stage in which the radicle was at a 45° angle from the central axis of the cotyledons. The subsequent developmental stages were 14, 17, and 20 DAP for both accessions. For biomass analysis, siliques at 30 DAP were senesced and therefore considered mature.

### Fatty Acid Extraction and Derivatization

Fatty acids were extracted and methylated in accordance with Cocuron et al. (2014). Combined extracts were dried under nitrogen gas at 60°C prior to methylation with 2.5% (v/v) sulfuric acid in methanol at 80–85°C for 90 min. After methylation, the reaction was quenched with 250 μl of a 5% (w/v) sodium hydrogen sulfate solution. The organic phase containing the fatty acid methyl esters (FAMEs) was diluted 5-fold with hexane in a 2 ml autosampler vial prior to GC–MS analysis.

### GC–MS Quantification of Fatty Acids

FAMEs were quantified in accordance with Tsogtbaatar et al. (2015) using an Agilent 6890N gas chromatograph (Agilent Technologies, Santa Clara, CA) coupled to a Agilent 5975B mass selective detector. An Omegawax 250 capillary column (30 m × 0.25 mm × 0.25 μm; MilliporeSigma, Burlington, MA) was used to separate FAMEs at a constant flow rate of 1.4 ml min^−1^ using helium as the carrier gas. The initial temperature was 120°C and held for 30 s, followed by an increase to 245°C for the remaining 9.5 min at a rate of 100°C min^−1^. The injection temperature was set at 225°C, with the injection mode set to a split ratio of 10:1. Mass spectra were acquired using electron impact ionization in positive ion mode, with ion source and interface set to 240 and 150°C, respectively. Acquisition and integration of GC–MS data was carried out *via* MSD ChemStation E.02.02.1431 (Agilent Technologies, Santa Clara, CA).

### Protein Extraction and Quantification

Total proteins were extracted from the pellet remaining from the FA extraction with a 20 mM Tris, 150 mM NaCl, and 1% SDS buffer in accordance with Cocuron et al. (2014) to obtain a final 1.5 ml volume of extract. Protein content was then quantified colorimetrically *via* the DC Protein Assay (Bio-Rad, Hercules, CA) in a Bio-Rad SmartSpec Plus spectrophotometer at 750 nm.

### Metabolite Extraction

Extraction of soluble metabolites was carried out according to previous research (Alonso et al., 2010; Cocuron et al., 2014) using boiling deionized water. Samples were then incubated in boiling water for a total of 10 min and ultimately passed through a 0.22 μm filter into a 15 ml conical tube on ice. The remaining pellet was rinsed with ice cold water, vortexed, and centrifuged again prior to passing through the same filter as the first supernatant. Ice-cold water was then used to remove any remaining metabolites in the syringe and filter. Lyophilized filtrate was resuspended in ice-cold water and passed through a 0.22 μm Nanosep filter (Pall Corp., Port Washington, NY) to use for sugar and sugar alcohol quantification, while the remaining volume was loaded onto a 3 kDa Ultracel filter (MilliporeSigma, Burlington, MA). All extraction steps were also performed with ^13^C-glycine, ^13^C-glucose, and ^13^C-fumarate internal standards without sample in order to calculate correction factors to control for errors during extraction or sample preparation.

### LC–MS/MS Quantification of Metabolites

Sugars and sugar alcohols, amino acids, organic acids, and phosphorylated compounds were quantified as previously described using programs described (Alonso et al., 2010; Koubaa et al., 2013; Cocuron and Alonso, 2014; Cocuron et al., 2014). For sugar and sugar alcohols, extracts were diluted in a 60:40 acetonitrile:water solution prior to injection onto a Shodex Asahipak NH2P-50 2D column and a NH2P-50G 2A guard column (Showa Denko K. K., Tokyo, Japan) followed by separation *via* an acetonitrile and water gradient (Cocuron et al., 2014). Mass spectra were obtained *via* turbo-spray ionization at 4,500 V in negative ionization mode, multi-reaction monitoring (MRM) mode with a dwell time of 100 msec for each transition. For amino acids, extracts were diluted with an HCl solution and water such that the final concentration in the autosampler vial was 1 mM HCl. Samples were incubated at 15°C prior to injection onto a Hypercarb column (Thermo Fisher Scientific, Waltham, MA) and were separated *via* a gradient of acetonitrile with 0.1% formic acid and water with 0.1% formic acid. Mass spectra were obtained at 2,500 V in positive ionization mode, MRM mode with a dwell time of 35 msec for each transition. For organic acids and phosphorylated compounds, extracts were diluted with HPLC-grade water and kept at 4°C prior to injection onto an IonPac AS11 column (Dionex, Sunnyvale, CA) with an IonPac AG11 guard column. A gradient of 0.5 mM KOH to 75 mM KOH was used to separate metabolites, with mass spectra in negative ionization mode, MRM mode. All metabolite data was acquired and integrated *via* Analyst (SCIEX, Framingham, MA). All samples were run using a 1,290 Infinity II ultra-high-performance liquid chromatograph (Agilent Technologies, Santa Clara, CA) coupled with a QTRAP 6500+ mass spectrometer (SCIEX, Framingham, MA). Data acquisition and quantification was carried out using Analyst v1.7 with HotFix 3 (SCIEX, Framingham, MA).

### RNA Extraction

RNA was extracted in accordance with Horn et al. (2016), with modifications. Approximately 1,000 μg of fresh embryo tissue was manually homogenized in a 2 ml microcentrifuge tube with plastic pestles directly in liquid nitrogen. A 600 μl volume per sample of extraction buffer of 2% CTAB, 2 M NaCl, 100 mM Tris (pH = 8), 25 mM EDTA (pH = 8), 3% PVP, 0.5 g L^−1^ spermidine, and 3% 2-sulfanylethan-1-ol that was preheated to 65°C was used for extraction. A 40 μl volume of 3.2 M sodium ethanoate (pH = 5.5), 100 μl of RNA-grade glycogen, and 800 μl of ethanol were added to each aliquot prior to a 12 h precipitation at −20°C. Each aliquot was then centrifuged at 21,000 × *g* for 1 h at 4°C, immediately placed on ice, and supernatant was discarded. A total of three 70% ethanol washes were carried out on the resulting pellet prior to being dried and dissolved in RNase-free water. A lithium chloride precipitation was then performed at −20°C for 12 h. Three more ice-cold ethanol washes were then carried out as previously stated, pellets were dried, and each sample’s pellet was dissolved in RNase-free water. Samples were stored at −80°C until further analysis.

### RNA Quality Confirmation and RNAseq

Analysis of the quality and quantity of RNA extracts was performed at the University of North Texas Genomics Center using an Agilent 2100 bioanalyzer (Agilent Technologies, Santa Clara, CA) and QUBIT 3.0 fluorometer (Invitrogen, Carlsbad, CA), respectively. All samples were confirmed to have RNA integrity numbers of ≥9. Samples were shipped overnight on dry ice to the location where RNAseq was performed using DNBseq Technology: NGS 2.0 (BGI, Cambridge, MA).

### Quality Control and Mapping of RNAseq Data

A total of 2,259 million sequences of raw data were generated with an average of 37.65 million reads per sample. A raw data quality check was carried out using FastQC (Andrews, 2010) and data was deposited online (NCBI Accession: PRJNA808106). Clean reads were then mapped to the pennycress reference genome (Dorn et al., 2015) using HISAT v2.1.0 (Kim et al., 2015) with default parameters. The mapped reads were then sorted and converted to .bam files using Samtools v1.9 (Li et al., 2009). Each aligned sample had an average mapping of 97%. The R package FeatureCounts v1.5 (Liao et al., 2014) was used to generate raw count data, and StringTie v2.1.1 (Pertea et al., 2016) was used to calculate transcripts per million (TPM). All genes were paired with respective *Arabidopsis thaliana* homologs for annotation and analysis using Araport (Krishnakumar et al., 2015; Pasha et al., 2020; Navarrete et al., submitted).

### Transcriptomics Data Processing

The resulting raw count data from RNAseq were transformed using DeSeq2 v1.30.1 (Love et al., 2014) to perform PCA as previously described (Gerst and Hölzer, 2018). A pool of differentially expressed genes (DEGs) was created using a DESeq analysis, which was carried out within each developmental stage. Pre-filtering was carried out by removing genes with an average of less than 5 TPM across all samples. All fold-changes were calculated by using HO in relation to LO. Genes which returned with padj ≤0.05 and a log_2_-fold change (log_2_FC) ≥ |1| were considered significant, with upregulated genes returning a logFC ≥1 and downregulated genes returning a log_2_FC ≤ −1. Adjusted values of *p* were corrected using the false discovery rate (FDR). Shrinkage of log_2_FC estimates (Zhu et al., 2019) was carried out to improve interpretability of MA plots (Supplementary Figure S2). The “rlog” function in DESeq2 was performed with a transformation not blinded by the experimental design for all downstream analyses, in accordance with the suggested workflow of the package (Love et al., 2014). Genes that maintained a statistical significance at all developmental stages were functionally profiled according to KEGG pathway membership *via* g:Profiler (Raudvere et al., 2019). Unless otherwise stated, all genes are referred to by their *A. thaliana* homolog, but also identified with their *T. arvense* number when applicable to show unique reads from our RNAseq dataset. Functional classification of DEGs based on gene ontology (GO) terms were carried out *via* PANTHER GO-Slim using the GO biological process (GO:BP) database (Mi and Thomas, 2009; Mi et al., 2021). Visualization of the DEG GO:BP profile was performed using BiNGO (Supplementary Figure S4; Maere et al., 2005).

The protein sequences of the 950 DeSeq-derived DEGs were incorporated into DeepLoc v.1.0 (Almagro Armenteros et al., 2017). This software uses recurrent neural networks (RNNs) to classify proteins in one of ten potential subcellular localizations (nucleus, cytoplasm, extracellular, mitochondrion, cell membrane, endoplasmic reticulum (ER), chloroplast, Golgi apparatus, lysosome/vacuole, and peroxisome; Supplementary Data Sheet S1). The cutoff value for considering a prediction was a likelihood higher than 0.7.

### Integration of Metabolomics and Transcriptomics Data

A joint pathway analysis was performed using MetaboAnalyst 5.0 to combine transcriptomics and metabolomics data in order to detect pathway enrichment and analyze pathway topology at 20 DAP (the maximum point of divergence in FA content for both accessions prior to the cessation of metabolic activity; Chong et al., 2018). A hypergeometric test was used for the enrichment analysis, relative betweenness centrality was used for the pathway topology, and “omics” integration was carried out by combining values of *p via* equally-weighted *Z*-tests (Chong et al., 2019). Pathways that returned with a *p*_adj_ ≤ 0.05 and pathway impact value ≥0.2 were considered significant. All returned pathways were from the KEGG database (Kanehisa and Goto, 2000; Kanehisa, 2019; Kanehisa et al., 2021).

### Coexpression Analysis

The “psych” package in R was used to calculate Pearson correlations of all rlog-transformed DEGs (Revelle, 2018). The correlation matrix was then filtered to include DEGs with >0.9 correlation and *p*_adj_ < 0.01. Further network statistics including degree and betweenness centrality were calculated using the “igraph” package in R to assist with visualizations (Csardi, 2014). The coexpression network was imported into Cytoscape v3.8.0 for all network visualization. Over-representation analysis (ORA) was run using the GO:MF, GO:BP, GO:CC, and KEGG database. ORA was carried out using g:Profiler using a threshold of 0.10. General parent GO terms (response to stimulus, metabolic process, binding, etc.) and redundant terms are omitted for conciseness; full results are in Suppl. Info. CytoHubba was used on the full DEG coexpression network to identify the top 10 most important hub genes based on the maximal clique centrality algorithm (Chin et al., 2014). Separate subnetworks were made for the top upregulated and downregulated hub genes, with first neighbors of each hub gene organized by membership in over-represented GO/KEGG terms. Genes not present in any overrepresented terms were organized according to the following functional categories: 1) lipid droplet (GO:0005811), lipid metabolic process (GO:0006629), or fatty acid metabolic process (GO:0006631); 2) central carbon metabolism (KEGG:00010,00020,00030,00500); 3) Biosynthesis of amino acids (KEGG:01230) or amino acid metabolism (KEGG:00250–00400); 4) electron transport chain (GO:0022900); and 5) peroxisome (GO:0005777). Genes with unknown function were also grouped. All other known genes were placed in the most common GO or KEGG term for visualization if not placed in any previously mentioned terms.

### MS Imaging and Confocal Microscopy

Pennycress seeds were prepared and processed for MS imaging as described by Sturtevant et al. (2021). Dry seeds were embedded into 10% gelatin, frozen at −80°C for 24 h, and then transferred to −20°C for 2–3 days prior to cryo-sectioning. Seeds were oriented within the gelatin so that medial-cross sections could be taken, giving both embryonic axis and cotyledonary tissues. Sections of 30 μm thickness were taken using a cryo-microtome (CM1950, Leica Biosystems, Germany) set at −16°C, followed by lyophilization for 3 h to remove moisture due to condensation. Sections that were intact and showed no cosmetic defects under light microscopy were chosen for MALDI-MS imaging analysis. Tissue sections chosen for MALDI-MS imaging were coated with 2,5-dihydroxybenzoic acid (DHB) by sublimation using a protocol adapted from Hankin et al. (2007). Matrix coated sections were then analyzed by MALDI-MS imaging with a MALDI-LTQ-Orbitrap-XL mass spectrometer (ThermoScientific). The MALDI source parameters were set to: 12 μJ/pulse, 10 laser shots per step, and raster step size of 40 μm. The orbitrap mass analyzer parameters were set to detect ions from 500 to 1,200 *m/z* at a set resolution of 60,000. Imaging data was processed using the open source software Metabolite Imager (Horn and Chapman, 2014).

The embryos were processed for Airyscan imaging, after staining of LDs with BODIPY 493/503 (Invitrogen) as previously described (Cai et al., 2015; Gidda et al., 2016). Micrographs of dry seeds were acquired with a Zeiss LSM710 microscope fitted with Airyscan confocal superresolution and the MBS 488 beam splitter using an Apochromat 63×/1.4 oil DIC objective lens (Carl Zeiss Inc., Germany). Excitation and emission signals of the BODIPY stained LDs were collected as single optical section images (z-sections) of cotyledons and embryonic axis cells. Airyscan images were processed using Zeiss Zen blue software.

## Results and Discussion

### Developing HO Embryos Produce Oil More Efficiently on a Dry Weight Basis and Accumulate More Erucic Acid in Cotyledons

At maturity, the total FA percentage on a per weight basis was 41% in HO embryos, whereas in LO it was 32% (*p* = 0.0132; Table 1). On a per embryo basis the total quantity of FA was not statistically significant between the two accessions; the higher dry weight in LO per embryo (*p* = 0.0051) was matched by a significantly greater total protein content (*p* = 0.037; Supplementary Figure S1). Increased dry weight in LO is thus at least conceivably explained by a higher production of total protein, as HO is able to produce a similar FA content while producing less biomass per embryo. A clear divergence in the biomass profile started around 17 DAP, indicating a potentially critical point in assessing differences between the two accessions. All FA species except for palmitic acid (C16:0) exhibited a significant difference between the two accessions, however the magnitude of these differences did not exceed 4% of the total composition in any case. Notably, long chain fatty acid content was higher in HO embryos (54% vs. 51% in LO). Erucic acid comprised 40% of the fatty acid composition in HO embryos compared to 36% in LO embryos. This is a modest difference compared to the range reported in previous research across wild populations, displaying a range of 30 to 39% erucic acid (Phippen and Phippen, 2013). Soluble sugars and sugar alcohols made up a significantly larger portion of LO embryos (14%) in comparison with HO embryos (11%; *p* = 0.0127). Starch content was negligible in both accessions (<0.1%).

**Table 1:** Total biomass composition (% composition and % w/w) per embryo in mature pennycress embryos of HO and LO accessions.

Component		HO		LO		Value of *p*
		Mean	SD	Mean	SD
Fatty acid composition	% C16:0	3.184	0.076	3.272	0.117	0.250
	% C18:0	**0.598**	0.033	**0.831**	0.114	**0.00775**
	% C18:1	**13.691**	0.376	**18.470**	0.316	**1.19E-06**
	% C18:2	**19.480**	0.150	**17.145**	0.239	**3.11E-06**
	% C18:3	**8.938**	0.171	**9.590**	0.171	**0.00673**
	% C20:1	**9.522**	0.227	**11.067**	0.329	**0.00025**
	% C20:2	**1.332**	0.084	**1.003**	0.080	**0.00126**
	% C22:1	**40.189**	0.309	**36.184**	0.450	**6.26E-06**
	% C24:1	**3.067**	0.267	**2.438**	0.098	**0.00441**
Fatty acid	% w/w	**41.084**	1.726	**31.789**	2.683	**0.0132**
Protein	% w/w	31.196	1.556	32.185	4.746	0.706
Starch	% w/w	0.024	0.009	0.030	0.008	0.339
Hemicellulose composition	% Arabinose	**30.285**	1.432	**37.148**	1.908	**0.00120**
	% Galactose	13.304	1.505	13.897	1.132	0.552
	% Glucose	12.996	1.103	11.934	1.492	0.296
	% Mannose	**22.791**	1.413	**17.619**	3.255	**0.0268**
	% Xylose	20.625	0.818	19.402	1.692	0.240
Hemicellulose	% w/w	1.693	0.362	1.267	0.281	0.112
Cellulose	% w/w	0.526	0.126	0.343	0.169	0.134
Soluble sugar and sugar alcohol composition	% Arabitol	**0.010**	0.002	**0.023**	0.005	**0.00180**
	% Fructose	**0.033**	0.007	**0.018**	0.004	**0.00546**
	% Galactinol	0.241	0.046	0.203	0.043	0.271
	% Hexitols	0.016	0.001	0.022	0.005	0.0528
	% Glucose	4.433	0.240	4.283	0.219	0.389
	% Inositol	**0.056**	0.006	**0.041**	0.004	**0.00577**
	% Raffinose	**1.160**	0.204	**0.625**	0.020	**0.00196**
	% Stachyose	**3.762**	0.434	**1.789**	0.115	**0.000120**
	% Sucrose	**90.251**	0.767	**92.976**	0.343	**0.00064**
	% Tetrols	**0.037**	0.006	**0.021**	0.002	**0.00269**
Soluble sugars and sugar alcohols	% w/w	**10.651**	1.457	**14.455**	1.607	**0.0127**
	Total recovery	86.928		77.354		

SD, standard deviation of the mean. Value of *p* corresponds to *t*-test results assuming equal variance with 4 biological replicates. Fatty acid (FA), hemicellulose, and soluble sugar and sugar alcohol composition indicate the percentage of total content for each individual class of compound. FA data was quantified using C17:0 internal standard. Biomass components in mature embryos were subjected to *t*-tests assuming equal variance to compare each accession. Bold text indicates significance at *p* < 0.05.

**Figure 1:**
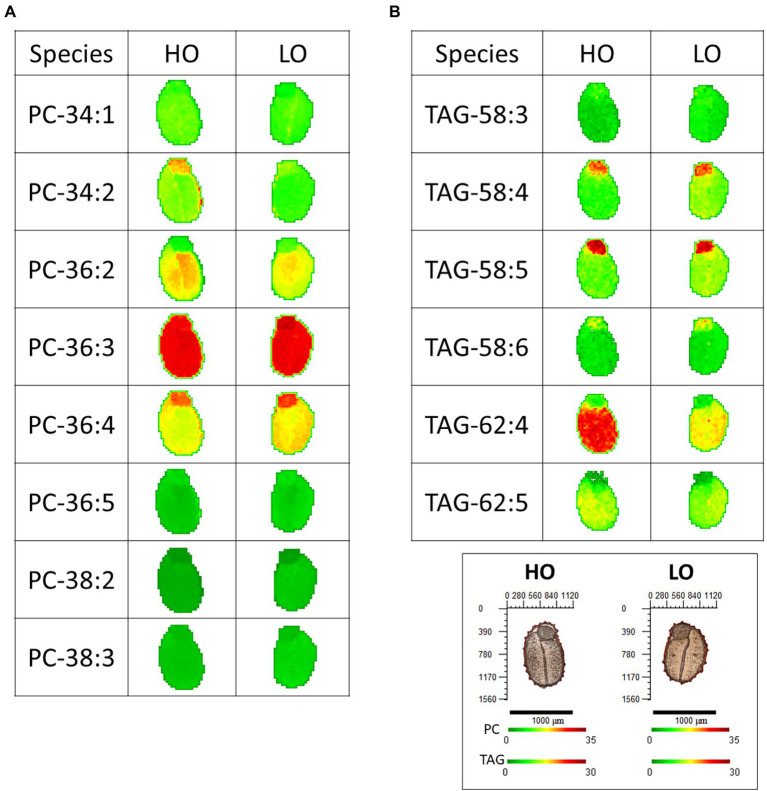
Relative abundance of **(A)** phosphatidylcholine (PC) and **(B)** triacylglycerol (TAG) species in high oil (HO) and low oil (LO) accessions of pennycress. Scale bar is 1,000 μm. Heat scale is normalized to the same intensity across species and accessions within each lipid class to allow for direct comparison. Imaging data was processed using Metabolite Imager (Horn and Chapman, 2014) and plotted as mol% using a colorimetric scale from green (low) to red (high). Images for PC are of the [M + H]^+^ adducts, while TAG are of the sum of [M + Na]^+^ and [M + K]^+^ adducts.

The distributions and relative amounts of phosphatidylcholine (PC) and TAG in embryos of both genotypes were visualized by MALDI-MS imaging, and representative images of selected molecular species are shown in Figure 1. PC is an intermediate in the desaturation and modification of acyl chains for TAG assembly, so these lipid classes often display precursor-product relationships. HO and LO genotypes displayed similar distributions and relative proportions of most PC molecular species, although heterogeneous distributions were evident within the embryos (between the cotyledons and the embryonic axis) for some PC species, such as PC 36:2 and PC 36:4 (Figure 1A).

The spatial distribution of TAG molecular species in embryos were similar between the two genotypes (Figure 1B). There were species that were notably concentrated in the embryonic axis (e.g., TAG- 58:4 and TAG 58:5). In contrast, erucic-acid-containing TAG molecular species appeared to be mostly restricted to cotyledons in both genotypes (TAG 62:4 and TAG 62:5), indicating a spatial distinction for FA elongation in the embryos. The HO genotype appeared to have relatively higher amounts of TAG 62:4 compared to the LO genotype (Figure 1B), consistent with the higher amounts of erucic acid in these seeds (Table 1). Taken together it appears that the HO genotype more readily produces very long chain (>18°C) monounsaturated FAs and incorporates them into the storage form of TAGs, and that this elongation occurs most predominantly in the cotyledons of the embryo. This is strongly supported by previous research in Brassicaceae, where in addition to pennycress, both *Brassica napus* and *Camelina sativa* are shown to be enriched for longer-chain (≥20°C) fatty acid species in the cotyledonary tissue compared to that of the embryonic axis (Horn et al., 2013; Lu et al., 2020; Romsdahl et al., 2021).

### Metabolomic and Transcriptomic Profiles Show Divergence Between Lines

**Figure 2:**
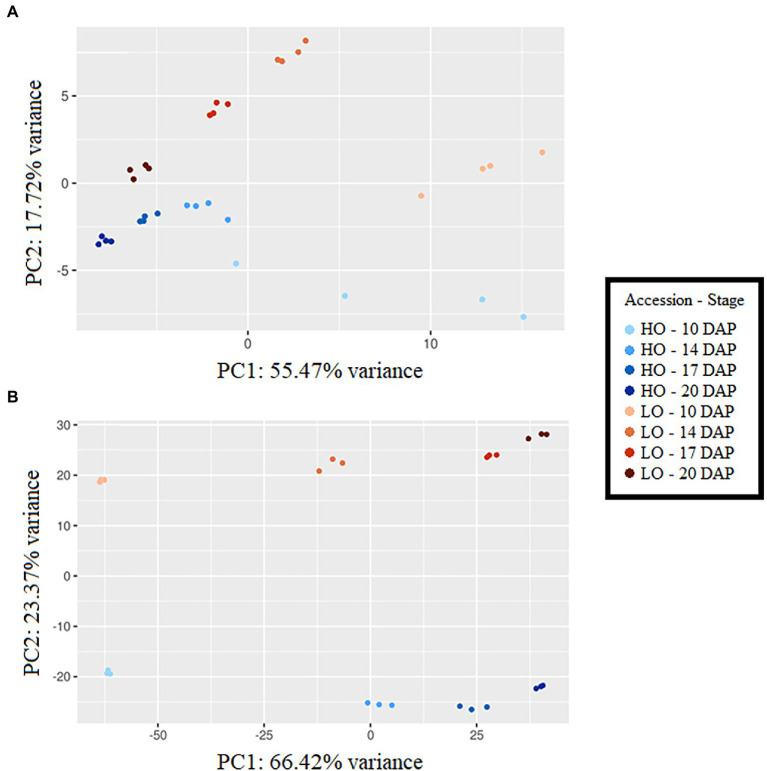
Principle component analysis using metabolomic dataset **(A)** and transcriptomic dataset **(B)**, DAP, days after pollination; HO, High oil accession; LO, Low oil accession. Principal component analysis was carried out on log-transformed and auto-scaled metabolite concentrations expressed in pmol mg dry weight (DW)^−1^ using PCAGO (Gerst and Hölzer, 2018).

**Figure 3:**
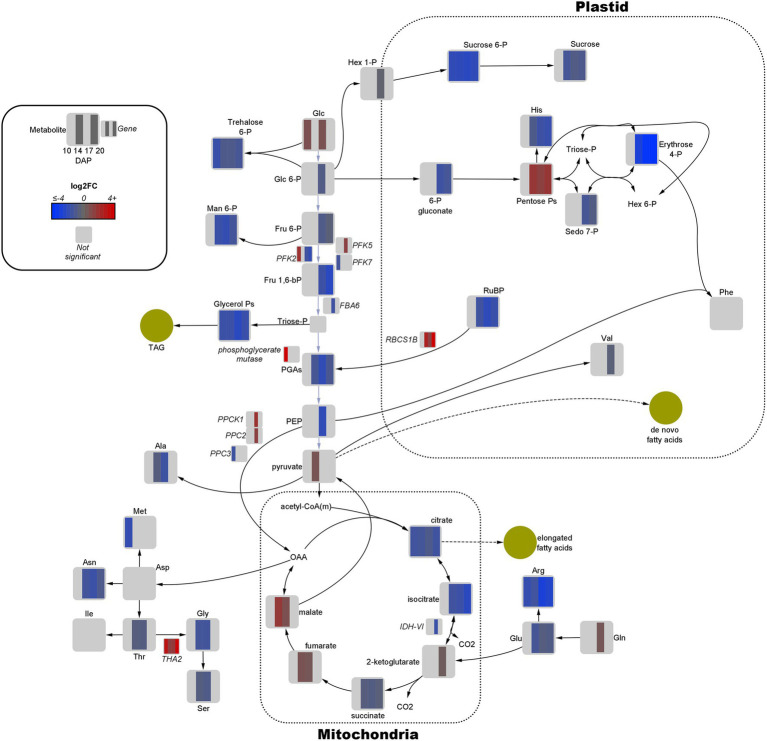
Fold-change of differentially expressed genes and significant metabolites involved in central carbon metabolism in high oil (HO) and low oil (LO) accessions of pennycress. Data are in log_2_FC in HO with respect to LO. Solid lines indicate single reactions between nodes, whereas dotted lines indicate the presence of more than one reaction. Yellow circles indicate corresponding fatty acid or lipid pools. –bP, bisphosphate; -P, −phosphate; acetyl-CoA(m), mitochondria-localized acetyl-CoA; Ala, alanine; Arg, arginine; Asn, asparagine; Asp, aspartate; CO_2_, carbon dioxide; FBA, fructose bisphosphate aldolase; Fru, fructose; Glc, glucose; Gln, glutamine; Glu, glutamate; Gly, glycine; Hex, hexose; His, histidine; IDH = isocitrate dehydrogenase; Ile, isoleucine; Man, mannose; Met, methionine; OAA, oxaloacetate; PEP, phosphoenolpyruvate; PFK, phosphofructokinase; PGAs, phosphoglycerates (both 2- and 3-); Phe, phenylalanine; PPC, phosphoenolpyruvate carboxylase; PPCK, phosphoenolpyruvate carboxylase kinase; RBCS1B, Rubisco small subunit; RuBP, ribulose 1,5-bisphosphate; Sedo, sedoheptulose; Ser, serine; THA, threonine aldolase; Thr, threonine; Val, valine.

Principal component analyses (PCAs) showed clear separation by accession and developmental stage using metabolomic and transcriptomic datasets (Figure 2). In metabolomics and transcriptomics datasets, principal component 1 (PC1) generally separated developmental stages, accounting for 55.47 and 66.42% variance, respectively. PC2, explaining 17.72 and 23.37% of the variance in metabolomics and transcriptomics datasets, respectively, was the primary separator of accessions. Time-series metabolite quantities between HO and LO genotypes were placed into four clusters (Supplementary Figure S3). Clusters 1 and 2 notably consisted of the majority of sugar phosphates, all basic and most polar amino acids, 3-carbon glycolysis intermediates (phosphoglycerates, phosphoenolpyruvate), and TCA intermediates upstream of isocitrate dehydrogenase (Figure 3). These metabolites were consistently higher in LO embryos throughout development. Cluster 3, encompassing metabolites that were constantly higher in HO, consisted of the main carbon sources glucose and sucrose, as well as key cell wall precursors. Specialization of metabolite investment thus appears to be a phenomenon associated with the differences between accessions.

### Alteration of Metabolic Pathways Spans Central Metabolism

**Figure 4:**
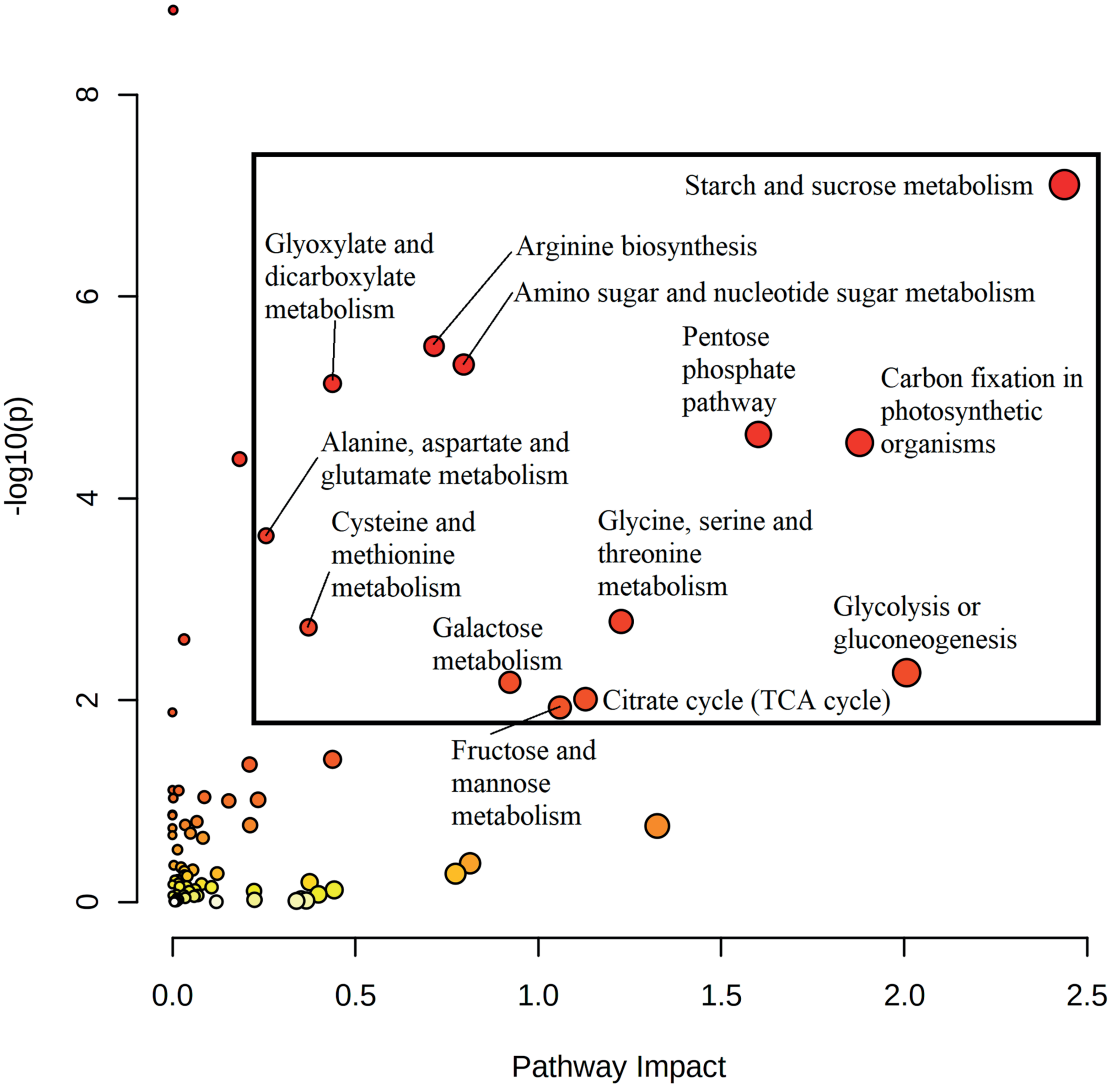
A joint pathway analysis was performed in order to detect pathway enrichment and analyze pathway topology at 20 DAP (the maximum point of divergence in FA content for both accessions prior to the cessation of metabolic activity). Node size indicates magnitude of pathway impact, whereas node color indicates enrichment analysis significance (with red indicating higher significance). Pathways that returned with a *p*_adj_ ≤ 0.05 and pathway impact value ≥0.2 were considered significant. All returned pathways were from the KEGG database (Kanehisa and Goto, 2000; Kanehisa, 2019; Kanehisa et al., 2021).

Quantitative joint pathway analysis identified all key parts of central carbon metabolism as being significantly enriched (*p*_adj_ ≤ 0.05) and impacted (impact factor ≥ 0.2), consisting of glycolysis, carbon fixation, the TCA cycle, and the OPPP (Figure 4). Of all significant pathways involved in central carbon metabolism, glycolysis was the most heavily impacted (Impact = 2.0067, *p*_adj_ = 0.0312). Key mapped metabolites and genes were downregulated in HO around the divergence point in the rate of oil accumulation. Specifically, pyruvate displayed a 1.6-fold increase in abundance in HO at 14 DAP, matched by a 1.7-fold increase in glucose at 17 DAP (Figure 3). The gene *phosphofructokinase 5* (*PFK5*), predicted by our modeling to be localized to the plastid, was upregulated 2.7-fold in HO at 17 DAP and may serve as a chief target in central carbon metabolism. The PFK step is known to be the rate-limiting step in glycolytic flux in previous research on *B. napus*, and its upregulation has been shown to explain the higher content of oil in oil palm vs. the sugar-accumulating date palm, presumably *via* increased pyruvate production (Junker et al., 2007; Bourgis et al., 2011).

Similar to glycolysis, there were only a few key metabolites in both the PPP and TCA cycle that were upregulated in HO at any developmental stage (Figure 3). Of note was the 2.8- to 3.7-fold increase in pentose phosphates from 10 to 17 DAP; coupled with a ≤2.4-fold decrease in 6-phosphogluconate content at 17 and 20 DAP, this strongly suggests a higher flow of carbon through the OPPP in HO embryos. As the OPPP is a major source of reductant (i.e., NADPH) and was shown to be mainly active in the cytosol in pennycress, increased total activity may contribute to FA elongation, and therefore to differential oil accumulation (Tsogtbaatar et al., 2020). Furthermore, malate, a carbon source for *de novo* FA biosynthesis, was 1.8- to 3.5-fold higher in HO embryos at 14 and 17 DAP, respectively. This directly correlates with the same increase in another 4-carbon TCA intermediate, fumarate. A significant 3.2-fold decrease in transcript levels of *isocitrate dehydrogenase catalytic subunit 6* (*IDH-VI*) expression at 17 DAP was observed in HO in the TCA cycle, which according to previous research on pennycress suggests a reduction of reverse flow through isocitrate dehydrogenase (Tsogtbaatar et al., 2020). This, along with a 1.9- to 2.8-fold reduction in citrate and a 2.3- to 4.9-fold reduction in isocitrate content, may suggest a reduced flow of carbon from fumarate/malate towards the 6-carbon metabolites of the TCA cycle. As such, this would preserve malate for potential carbon incorporation into *de novo* FA metabolism in the plastid. Interestingly, at 17 DAP there was a 2.8-fold increase in *phosphoenolpyruvate carboxylase 2* (*PPC2*) expression in HO embryos, which was simultaneous with a 4.1-fold increase in *phosphoenolpyruvate carboxylase kinase* (*PPCK1*) expression. This provides good evidence of both transcriptional (*via PPC2* expression) and post-translational (*via* increased abundance of PPCK1) upregulation of the flow of carbon through the anaplerotic pathway from phosphoenolpyruvate (PEP) to oxaloacetate (OAA), as PPCKs phosphorylate PPC which is generally associated with increased anaplerotic pathway flow (Nimmo, 2003).

The gene *RBCS1B*, encoding a Rubisco subunit, displayed 3-fold upregulation at 17 DAP, increasing all the way to an 1,176-fold increase at 20 DAP in HO. This in addition to up to a 5-fold decrease in ribulose 1,5-bisphosphate (RuBP) abundance at 17 DAP suggests a potentially higher degree of carbon fixation. An additional function of Rubisco of re-fixing plastidic CO_2_ lost *via* other metabolic processes has been demonstrated in pennycress embryos and other Brassicaceae, which provides additional C source for *de novo* FA synthesis (Schwender et al., 2004; Tsogtbaatar et al., 2020). The upregulation of Rubisco activity suggested by the increase of *RBCS1B* may allow HO to more efficiently reincorporate lost CO_2_.

In light of the current knowledge of pennycress, results were interpreted in order to determine if the unusual pathways previously found in pennycress were more active in HO vs. LO embryos. These pathways included: (1) high activity of the OPPP in the cytosol, (2) the reversibility of the isocitrate dehydrogenase that produces isocitrate from α-ketoglutarate; and (3) re-fixation of plastidic CO_2_ released by the NADP-dependent malic enzyme and pyruvate dehydrogenase *via* Rubisco (Tsogtbaatar et al., 2020). Our results demonstrate conflicted findings concerning which of these pathways are positively associated with oil production in HO. Specifically, isocitrate and citrate content as well as *TaIDH-VI* expression were lower in HO, suggesting that reversibility of isocitrate dehydrogenase is actually downregulated in HO (Tsogtbaatar et al., 2015, 2020). However, despite increased malate content in HO, no upregulation of genes encoding an NADP-dependent malic enzyme or pyruvate dehydrogenase were noted, which suggests that achieving improved oil content in pennycress is not necessarily limited by carbon incorporation into plastidic *de novo* FA *via* these canonical pathways.

In these results, only a suggested upregulation of the Calvin cycle and higher OPPP activity are consistent with previously published reasons that pennycress has improved carbon conversion efficiency compared to other Brassicaceae (Tsogtbaatar et al., 2015, 2020). Furthermore, improved glycolytic flux *via* increased *PFK5* expression, and enhanced anaplerotic pathway flux *via* increased *PPC2* and *PPCK1* expression may play a key role in HO to achieve higher % w/w oil (or less protein) content. The lack of evidence for increased malate flow into the plastid *via* NADP-dependent malic enzyme, and evidence that higher oil content is not due to reversible ICDH activity in HO therefore suggests that additional pathways or reactions are key to HO producing more oil than its LO counterpart.

### Threonine Aldolase Upregulation May Provide Carbon Sources for Oil Production

**Figure 5:**
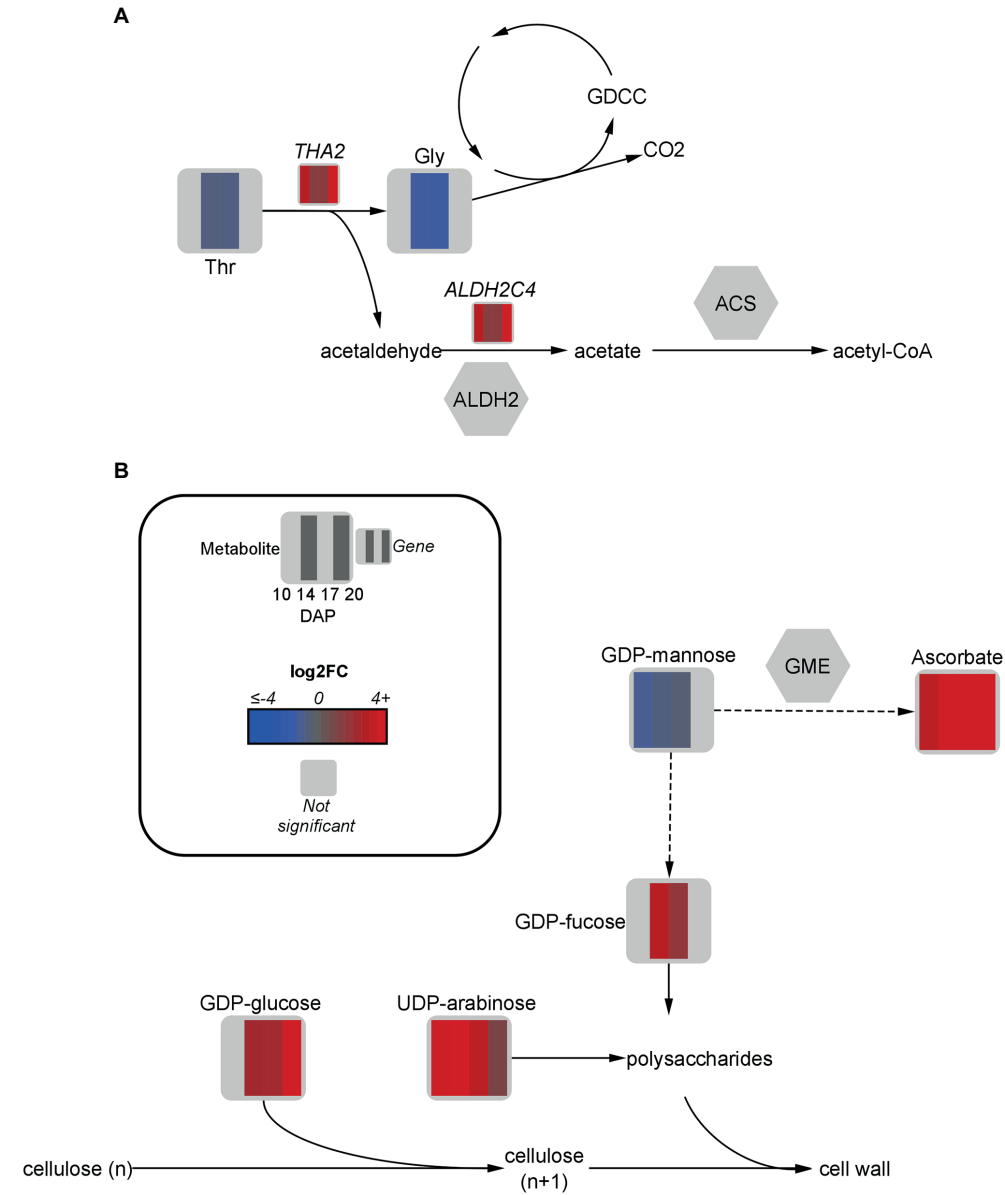
Fold-change of DEGs and significant metabolites involved in threonine metabolism **(A)** and cell wall metabolism **(B)** in high oil (HO) and low oil (LO) accessions of pennycress. Data are in log_2_FC in HO with respect to LO. Solid lines indicate single reactions between nodes, whereas dotted lines indicate the presence of more than one reaction. ACS, acetyl-CoA synthase; ALDH, aldehyde dehydrogenase; CO2, carbon dioxide; DXPS, deoxyxylulose phosphate synthase; GDCC, glycine decarboxylase complex; Gly, glycine; GME, GDP-D-mannose 3,5 epimerase; *n*, number of carbons in cellulose units; PDH, pyruvate dehydrogenase; TDP, thiamine diphosphate; THA, threonine aldolase; Thr, threonine.

The reduced protein accumulation in HO prompted investigation into amino acid metabolism. One of the most upregulated genes involved in central carbon metabolism was *THA2*, encoding a threonine aldolase with no specific predicted localization. Expression was significantly higher in HO at all developmental stages, and by 20 DAP a 17-fold increase in expression was observed (Figure 5A). In Arabidopsis and pennycress, significant research has established that *THA2* is generally unidirectional, resulting in the catabolism of threonine to glycine and acetaldehyde, under non-photorespiratory conditions (Jander et al., 2004; Joshi et al., 2006; Tsogtbaatar et al., 2020). As expected, a transient ~1.5-fold decrease in threonine content was observed in HO at 14 and 17 DAP. However, the same trend was observed with glycine (2.5-fold decrease). This provided evidence that accumulation of glycine was not the final result of upregulated *TaTHA2*, and that further flow of carbon through glycine and acetaldehyde was likely.

While multiple roles of threonine aldolases have been suggested in plants, a particular role of interest with regard to FA production is the conversion of its breakdown product acetaldehyde to acetyl-CoA *via* aldehyde dehydrogenases (ALDH) followed by acetyl-CoA synthase (Jander et al., 2004). This strategy serves as a pyruvate dehydrogenase bypass, allowing for carbon from aldehydes to be directly converted to acetate for acetyl-CoA production (Wei et al., 2009). One specific ALDH transcript, *ALDH2C4*, was upregulated 3-fold at 20 DAP, which is notable considering FA production continues in HO embryos after LO has generally ceased accumulation (Supplementary Figure S1A). In *A. thaliana*, ALDH2C4 is cytosolic and generally associated with phenylpropanoid metabolism (Wei et al., 2009). However, research has suggested it may also play at least a partial role in the pyruvate dehydrogenase bypass to funnel carbon towards acetyl-CoA, in addition to the activity of mitochondrial ALDH enzymes (Nair et al., 2004; Wei et al., 2009). Another route of aldehyde formation is *via* ethanolic fermentation by alcohol dehydrogenase (Wei et al., 2009). A cytosolic *ADH-like 1* gene was upregulated 4.5 to 12.4-fold in HO embryos from 10 to 17 DAP, suggesting a further source of acetaldehyde for acetyl-CoA production is occurring.

The glycine decarboxylase complex is another potential downstream use of threonine aldolase products, which upon metabolizing glycine releases CO_2_ (Igamberdiev et al., 2004). As previously mentioned, this CO_2_ can theoretically then be reincorporated *via* the reversible ICDH activity, or into OAA *via* phosphoenolpyruvate carboxykinase (Tsogtbaatar et al., 2020). Further research should be done to determine the flux of threonine-derived glycine and acetaldehyde through these pathways to incorporate carbon towards FA synthesis and elongation.

### Ascorbate and Nucleotide Sugar Metabolism Suggest Cell Wall Contributions

Among the most upregulated metabolites in HO from 17 to 20 DAP were the key cell-wall precursors UDP-arabinose (9.4- and 2.3-fold), GDP-glucose (5.2- and 278.2-fold, respectively), and GDP-fucose (3.8-fold at 17 DAP). Consistent with this upregulation was a slightly altered hemicellulose composition between each accession. Indeed, the accumulation of UDP-arabinose in HO was associated with a 19% reduction in arabinose content in the hemicellulose fraction of the cell wall (Figure 5B). Furthermore, a nearly 30% increase in mannose content in the hemicellulose fraction of HO was observed. The only developmental stages in which GDP-mannose, the precursor for hemicellulosic mannose, was significantly different between accessions was 10 and 14 DAP, where a 1.5 to 2-fold reduction was observed in HO (Brar and Elbein, 1972).

GDP-D-mannose 3,5 epimerase is an enzyme responsible for diverting GDP-mannose away from cell wall production and toward the production of ascorbate (Wolucka and Van Montagu, 2003; Gilbert et al., 2009). Interestingly, ascorbate was confirmed in HO at 8,135 and 11,656 pmol mg dry weight (DW)^−1^ from 17 to 20 DAP, respectively. However, ascorbate was below the limit of quantification in LO and in general has not been previously mentioned as present in pennycress. Ascorbate reduces membrane lipid peroxidation during stress which may preserve total lipid content in HO (Ebrahimian and Bybordi, 2012). Furthermore, ascorbate can act as an alternate electron donor in various areas of plant metabolism including that of embryos, and as such may contribute extra reductant for FA synthesis and/or modification (Mano et al., 2004; Tóth et al., 2009; Hoang et al., 2021). Ascorbate has also been shown to regulate amino acid synthesis, which may relate to the different findings on amino acid and protein content in HO (Gulyás et al., 2017). The novel finding of ascorbate accumulation, along with the altered hemicellulose composition, suggests a difference in cell wall metabolism or redox regulation exists between accessions.

**Figure 6:**
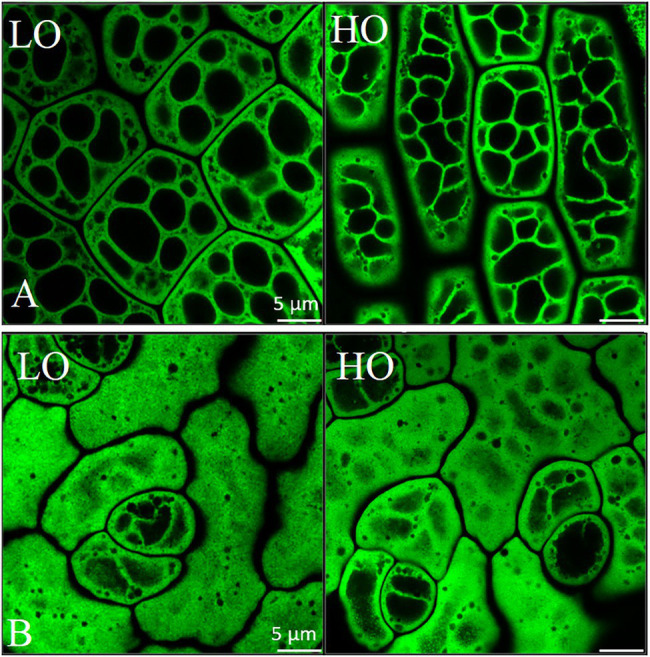
Airyscan imaging of lipid droplets in embryonic axis **(A)** and cotyledonary **(B)** tissue in mature HO and LO pennycress embryos. Scale is 5 μm. Mature seeds were imaged after staining of lipid droplets with BODIPY 493/503 (Invitrogen, Carlsbad, CA). Micrographs of dry seeds were acquired with a Zeiss LSM710 microscope fitted with Airyscan confocal superresolution and the MBS 488 beam splitter using an Apochromat 63×/1.4 oil DIC objective lens (Carl Zeiss Inc., Thornwood, NY). Excitation and emission signals of the BODIPY-stained lipid droplets were collected as single optical section images (z-sections) of cotyledons and embryonic axis cells. Airyscan images were processed using Zeiss Zen Blue software.

A slight upregulation of *BGLC1* (3.3- and 2.9-fold at 17 and 20 DAP, respectively) was observed in HO. This gene encodes a glycosyl hydrolase that is thought to be able to cleave glucose from xyloglucan, which may have implications on cellular morphology and development (Saffer, 2018; Rubianes et al., 2019). Consistently, a difference in the cellular morphology in the embryonic axis of HO can be seen when compared to LO, consequently affecting lipid arrangement in this organ (Figure 6A). A potential reduction in xyloglucan content at any point of embryo development may allow cleaved glucose to be redirected elsewhere. More research is necessary to determine if the glucose removed from xyloglucan has any fate in oil metabolism or energy production. The increased glycolytic flow suggested in HO could theoretically funnel this glucose to produce more pyruvate. Ultimately, total free sugar content (Table 1) was 33% lower in HO on a % w/w basis compared to LO, highlighting that sugar allocation in high oil pennycress is likely altered. The substantial difference in cell wall features between accessions, as well as their association with ascorbate production, suggests a potential avenue for future research on contributions to lipid metabolism.

### Downregulation of the Ubiquitin Proteasome System and Increased Lipid Droplet Stability Are Associated With Higher Oil Production

**Figure 7:**
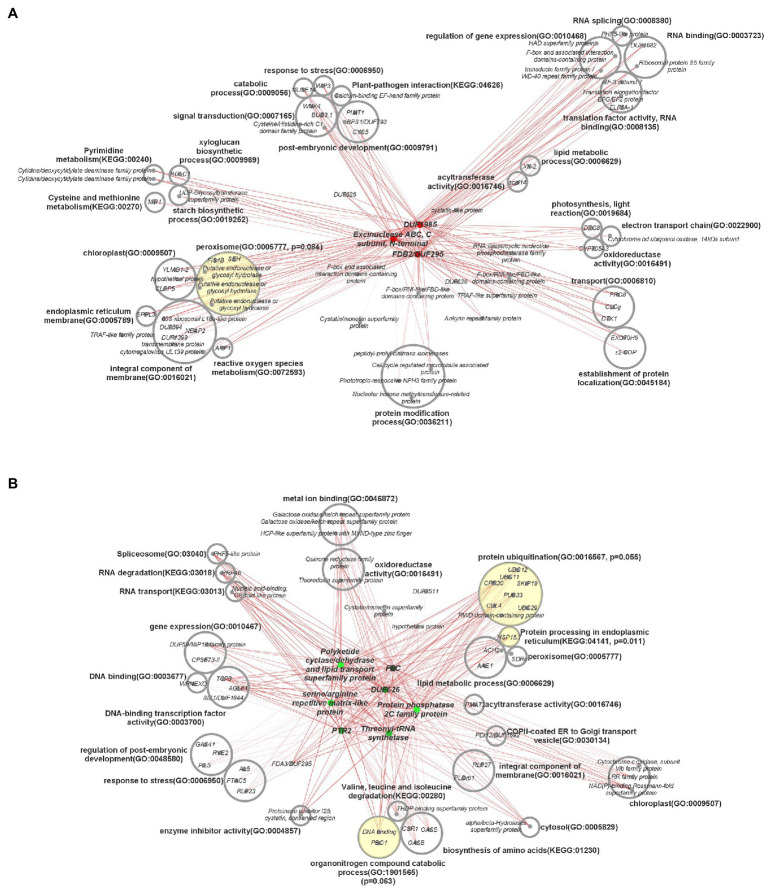
Central coexpression network of DEGs containing top 10 major hub genes and their respective first neighbors. **(A)** Three main hub genes that were upregulated in high oil accession, **(B)** seven main hub genes that were downregulated in high oil accession. Hub genes are in the center of each network, in color, and first neighbor genes are categorized by gene ontology terms. Circles with yellow shading indicate over-represented gene ontology terms, with *p*-values based on the false discovery rate, and their associated genes. DUF = gene with a domain of unknown function. Darkness of lines indicates correlation, with darker indicating higher correlation. ORA was carried out using a threshold of 0.10.

**Figure 8:**
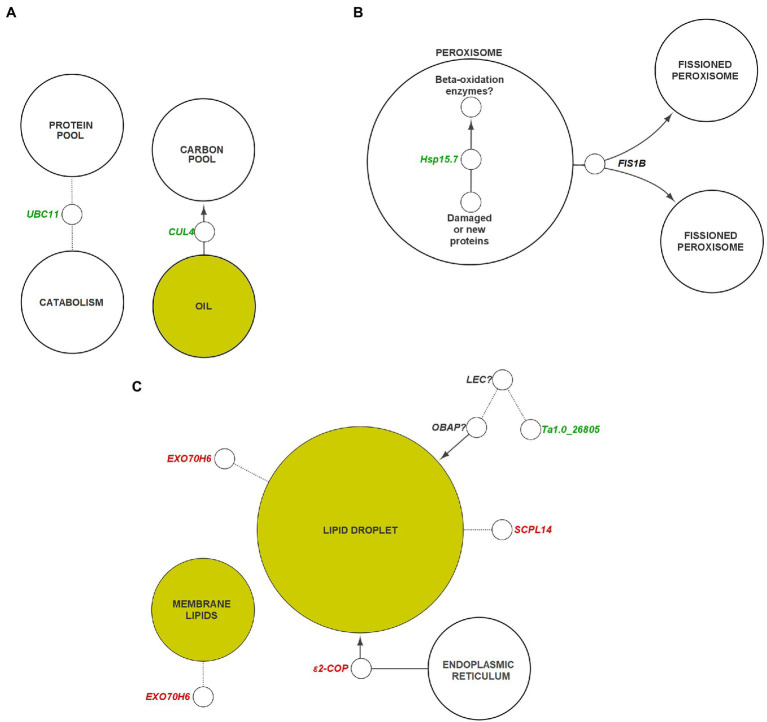
General pathways and components of plant metabolism containing potential targets for genetic engineering. **(A)** Ubiquitination, **(B)** peroxisome, **(C)** membranes and lipid droplets. Genes colored in green indicate those that are suggested to be negatively correlated with oil content, whereas genes in red indicate those that are positively correlated with oil content. Dotted lines indicate unconfirmed interactions between components/genes. Arrowheads indicate a gene or component has a net positive effect on the accumulation of their target. Genes with question marks are suggested targets that require specific identification within the respective gene family in *Thlaspi arvense*. Yellow nodes indicate lipid-related components. Italics indicate gene transcripts or corresponding protein could be interactor indicated by node. CUL, CULLIN; ε2-COP, epsilon2 coat protein; EXO70, exocyst subunit exo70; FIS, FISSION; Hsp, heat shock protein; LEC, leafy cotyledon; OBAP, oil body associated protein; SCPL, serine carboxypeptidase-like; UBC, ubiquitin-conjugating enzyme.

Interconnected transcriptional control of protein dynamics, amino acid metabolism, RNA/DNA modification, redox regulation, and lipid and FA metabolism are illustrated by coexpression analysis (Figure 7). Specifically, these results indicated a perturbation in genes associated with the stability of proteins, lipids, and lipid droplets (LD). Protein ubiquitination (*p*_adj_ = 0.055) and protein processing in the endoplasmic reticulum (*p*_adj_ = 0.011) were significantly over-represented connections to downregulated hub genes. In plants, the ubiquitin-proteasome system is associated with a diverse array of functions, particularly proteolytic action related to plant development, immunity, stress, and hormone response (Liu et al., 2020). Transcription of *UBC11*, associated in previous research with protein degradation in aging seeds (Figure 8A), was downregulated 150.1–739.3 fold throughout development in HO (Wang et al., 2018). This same trend was exhibited by both *UBC12* (17.4–278.2 fold decrease) and *UBC29* (16.4–634.7 fold decrease). Recent research has also suggested a link between cullin-RING ligases, enzymes critical to targeting proteins for ubiquitination, and lipid production in photosynthetic organisms. *Chlamydomonas reinhardtii* was shown to display a lower biomass per cell but higher total lipid content per cell when one of these ligases, *CULLIN4* (*CUL4*), was silenced using RNAi (Luo et al., 2021). This is highly consistent with our biomass results in pennycress, as *CUL4* was constitutively downregulated 4.3–5.7-fold throughout development. Taken together, the overall knockdown of a specialized component of the protein ubiquitination system appears to be related to oil production in pennycress.

HO embryos displayed a 5.4-fold increase in expression (17 DAP) of *FIS1B*, homologous with an *A. thaliana* gene which is noted to be directly responsible for peroxisome fission following elongation (Lingard et al., 2008; Zhang and Hu, 2008). Peroxisomes encapsulate a large portion of the system required for FA catabolism in plants (Wright and Bartel, 2020). Knockout of FIS1B activity has been shown to result in elongated, enlarged peroxisomes, while the inverse is true following insertion of *FIS1A* (Zhang and Hu, 2009; Kao et al., 2018). An overabundance of this protein in pennycress may therefore result in differential effects on FA catabolism (Figure 8B). Although no DEGs were detected that were directly involved TAG hydrolysis or β-oxidation (Supplementary Data Sheet S1), a reduction in the cell volume available for FA catabolism could theoretically shift the balance between oil synthesis and catabolism toward synthesis.

We examined the organization of lipid storage in cells of both genotypes by enhanced-resolution, confocal fluorescence microscopy. BODIPY 495/503 selectively stains neutral lipids in cells and here the arrangement of LDs, packed with neutral lipids, is visualized in cotyledons of both HO and LO embryos (Figure 6B). The cellular distribution of LDs in both genotypes was similar although the LDs may be more tightly packed together in HO. Transcriptomic contributions to the cellular organization of lipid storage are suggested by DEG results. The gene encoding the Coat Protein I (COPI) epsilon subunit, *ε2-COP*, was upregulated 3.3–6.2 fold throughout development (Figure 8C). The COPI complex is has been demonstrated to associate the LD with the ER, allowing for targeting of proteins to the LD membrane (Wilfling et al., 2014; Huang et al., 2019). Furthermore, a ≥ 81-fold increase throughout development in HO of *EXO70H6*, part of the largely uncharacterized exocyst complex, was found to affect the phospholipid composition in membranes, cell wall thickening, and overall recognition of lipids at the membrane surface (He et al., 2007). In *A. thaliana*, the loss of *serine-carboxypeptidase-like 41* (*SCPL41*), involved in protein turnover, has resulted in increased levels of membrane lipids (Chen et al., 2020). The observed 74–286 fold increase in *SCPL14* in HO may suggest that this isoform could interact with LDs or membrane lipids to facilitate the efficient subcellular organization of LDs (Figure 8C).

A polyketide cyclase/dehydrase and lipid transport superfamily protein, *Ta1.0_26805*, displayed high expression in LO throughout development, but was undetectable in HO. Previous research in *A. thaliana* showed it to be involved in a network that contains a mutual regulator/interactor (*LEC1*) of *OBAP1a*, a gene involved in LD stability (López-Ribera et al., 2014; Depuydt and Vandepoele, 2021). While there was no differential expression of the pennycress *LEC1* homolog, the lack of apparent *Ta1.0_26805* transcription in HO warrants investigation to determine if any interactions are present between it and another transcriptional regulator in pennycress. The discovery of a regulator similar in function to *LEC1* in pennycress might provide potential as a regulatory target for improving LD assembly or stability, however the possibility that there are sequence dissimilarities in *Ta1.0_26805* between accessions must also be considered. LEC1 is part of a regulatory network of transcription factors known as LAFL responsible for controlling seed maturation and accumulation of storage components (Santos-Mendoza et al., 2008; Verdier and Thompson, 2008). Interestingly none of the other genes of LAFL nor several known targets of these genes were differentially expressed in our data (Fatihi et al., 2016; Supplementary Data Sheet S1). Together these results suggest an enhanced system in HO for maintaining the access of biosynthetic enzymes to membranes and LDs, together with a reduction in a component of the ubiquitin-proteasome system.

### Comparative “Omics” Analysis Suggests Future Targets for Genetic Engineering

Oil production, from *de novo* FA synthesis to lipid packaging, is an integrated process and requires multiple approaches to assess its associated metabolism. This research illustrates evidence of several specific regions of plant metabolism that are perturbed during high oil production. Additionally, it displays evidence that previously-identified mechanisms that allow pennycress to produce oil more efficiently than fellow Brassicaceae are not necessarily targets for improving oil production within pennycress itself.

This research highlights four metabolic processes that appear to be positively associated with pennycress oil content: (1) carbon partitioning in central carbon metabolism that accumulates malate, pentose phosphates, increased flow through the glycolysis and anaplerotic pathway; (2) production of carbon precursors *via* threonine aldolase activity, either by pyruvate bypass and/or the glycine decarboxylase complex; (3) alteration of sugar incorporation into cell wall components, potentially *via* ascorbate metabolism; and (4) expression-level alteration of ubiquitination and LD organization. Augmentation of these cellular processes may provide lucrative genetic engineering targets for pennycress and other oilseeds as well as critical insights into the cross-talk between lipid metabolism and other pathways in plants in general.

## Data Availability Statement

The raw data presented in the study are deposited as Supplementary Material and in the https://www.ncbi.nlm.nih.gov repository, accession number PRJNA808106.

## Author Contributions

AA and EG designed the experiments. CJ, EO, TR, and AG performed the research. CJ, LTG, EO, TR, and AG performed data analysis and collection. CJ, LTG, EO, TR, AG, KC, EG, and AA performed data interpretation. CJ wrote the manuscript. LTG, EO, TR, AG, KC, EG, and AA assisted with manuscript preparation. All authors contributed to the article and approved the submitted version.

## Funding

This work was supported by the Department of Energy Office of Science, Office of Biological and Environmental Research (BER), grant # DE-SC0019233 and # DE-SC0020325.

## Conflict of Interest

The authors declare that the research was conducted in the absence of any commercial or financial relationships that could be construed as a potential conflict of interest.

## Publisher’s Note

All claims expressed in this article are solely those of the authors and do not necessarily represent those of their affiliated organizations, or those of the publisher, the editors and the reviewers. Any product that may be evaluated in this article, or claim that may be made by its manufacturer, is not guaranteed or endorsed by the publisher.

## Abbreviations

HO: High oil
LD: Lipid droplet
LO: Low oil
