# Supplementary material for: Effective Mechanisms for Improving Seed Oil Production in Pennycress (Thlaspi arvense L.) Highlighted by Integration of Comparative Metabolomics and Transcriptomics
Source: Front Plant Sci. 2022 Jul 14;13:943585. doi: 10.3389/fpls.2022.943585 (PMC9330397; doi:10.3389/fpls.2022.943585)
Supplement: Supplementary file 2 [file Data_Sheet_1.PDF]

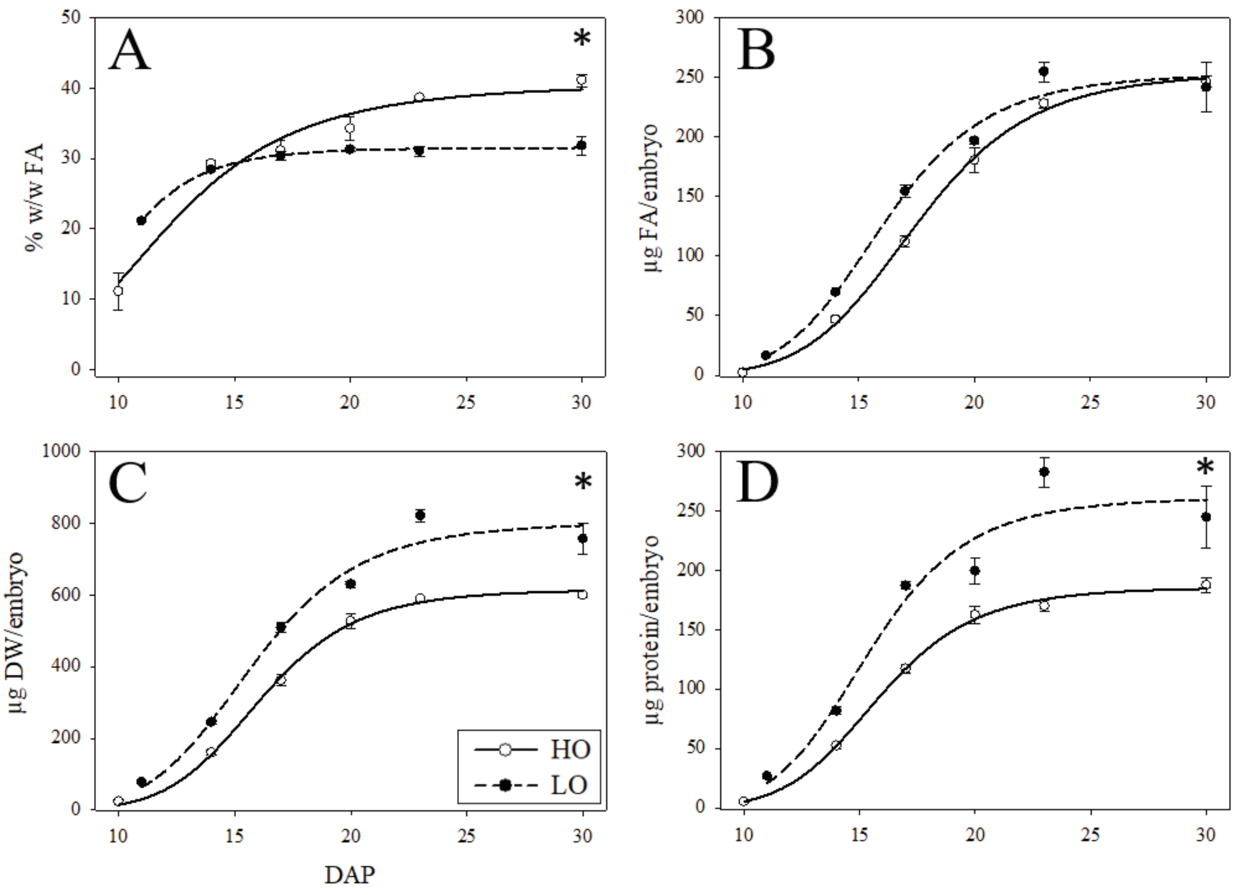

Figure S1. Biomass and fatty acid composition of developing high- and low-oil accessions of pennycress. A) percent fatty acid on a weight per embryo basis, B) fatty acid content per embryo, C) dry weight content per embryo, D) protein content per embryo. DAP = days after pollination; HO = High oil accession; LO = Low oil accession. Error bars represent standard error of the mean. FA data was quantified using C17:0 internal standard. All data are expressed as averages of 4 biological replicates. Asterisks indicate a significant difference at maturity with $p < 0.05$. Nonlinear regression was performed using a 3-parameter logistic sigmoidal function for all variables using SigmaPlot v12.3 (SPSS Inc., Chicago, IL).

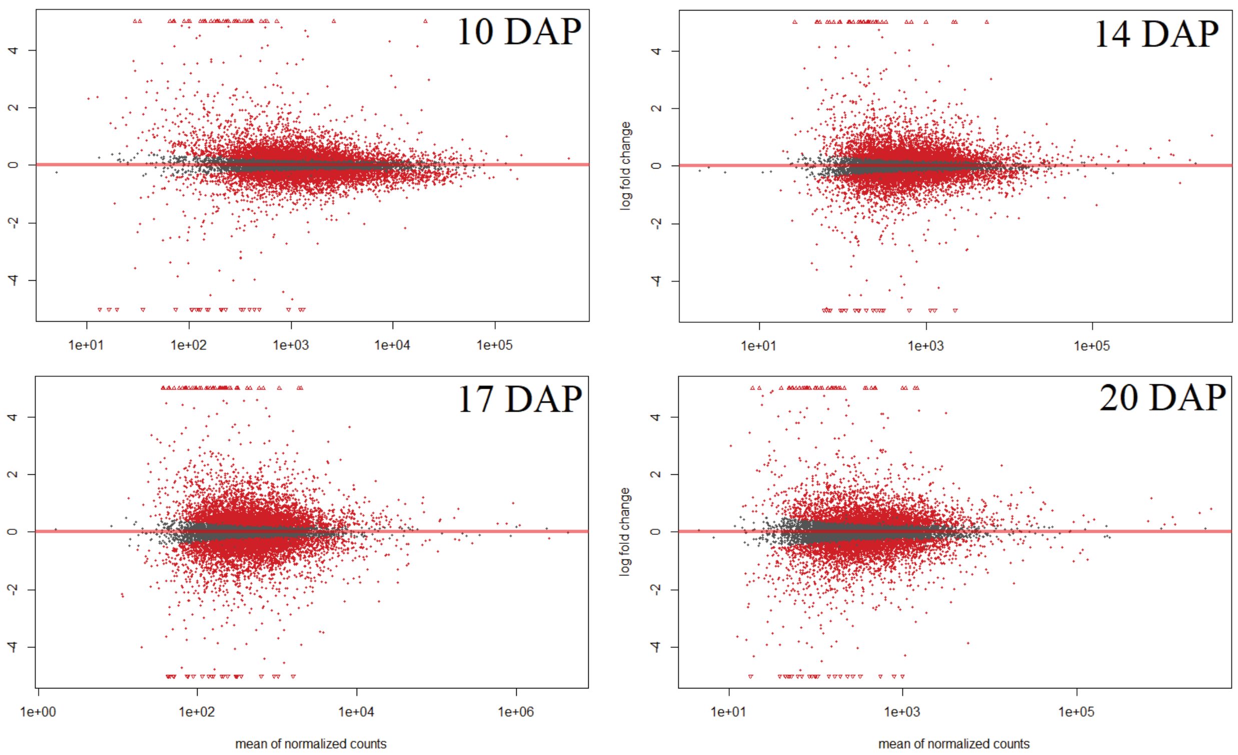

Figure S2. MA plots from DESeq2 detection of differentially expressed genes DEGs. A pool of DEGs was created using a DESeq analysis. Pre-filtering was carried out by removing genes with an average of less than 5 TPM across all samples. Genes which returned with $p_{adj} \leq 0.05$ and a log_2-fold change (log_2FC) $\geq |1|$ were considered significant. Shrinkage of log_2FC estimates (Zhu *et al.*, 2019) was carried out to improve interpretability.

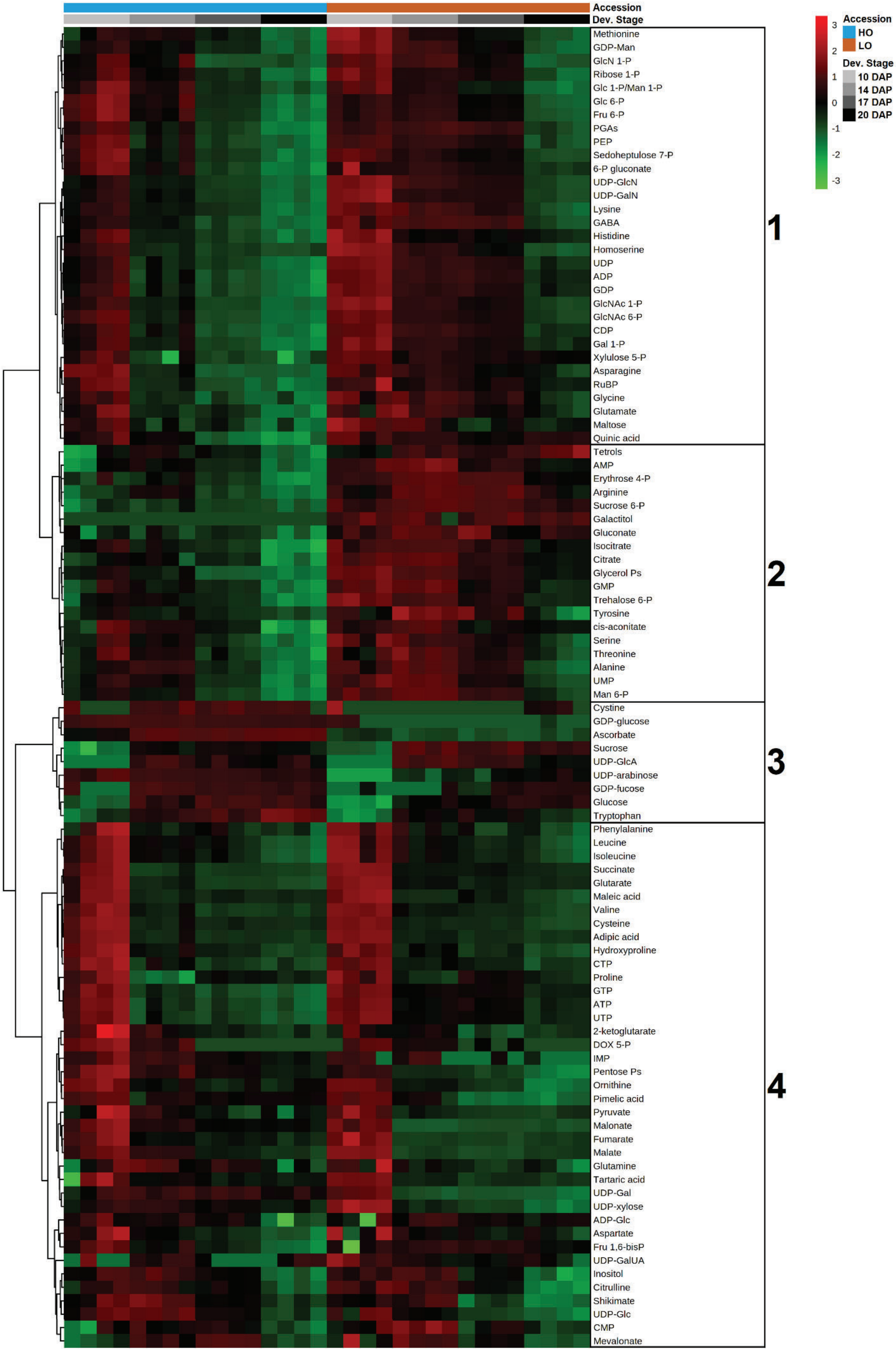
Accession
Dev. Stage
3
2
1
0
-1
-2
-3
Accession
HO
LO
Dev. Stage
10 DAP
14 DAP
17 DAP
20 DAP
Methionine
GDP-Man
GlcN 1-P
Ribose 1-P
Glc 1-P/Man 1-P
Glc 6-P
Fru 6-P
PGAs
PEP
Sedoheptulose 7-P
6-P gluconate
UDP-GlcN
UDP-GalN
Lysine
GABA
Histidine
Homoserine
UDP
ADP
GDP
GlcNAc 1-P
GlcNAc 6-P
CDP
Gal 1-P
Xylulose 5-P
Asparagine
RuBP
Glycine
Glutamate
Maltose
Quinic acid
Tetrols
AMP
Erythrose 4-P
Arginine
Sucrose 6-P
Galactitol
Gluconate
Isocitrate
Citrate
Glycerol Ps
GMP
Trehalose 6-P
Tyrosine
cis-aconitate
Serine
Threonine
Alanine
UMP
Man 6-P
Cystine
GDP-glucose
Ascorbate
Sucrose
UDP-GlcA
UDP-arabinose
GDP-fucose
Glucose
Tryptophan
Phenylalanine
Leucine
Isoleucine
Succinate
Glutarate
Maleic acid
Valine
Cysteine
Adipic acid
Hydroxyproline
CTP
Proline
GTP
ATP
UTP
2-ketoglutarate
DOX 5-P
IMP
Pentose Ps
Ornithine
Pimelic acid
Pyruvate
Malonate
Fumarate
Malate
Glutamine
Tartaric acid
UDP-Gal
UDP-xylose
ADP-Glc
Aspartate
Fru 1,6-bisP
UDP-GalUA
Inositol
Citrulline
Shikimate
UDP-Glc
CMP
Mevalonate
1
2
3
4

Figure S3. Heatmap of metabolite content in high oil (HO) and low oil (LO) pennycress accessions across development. Clustering analysis was performed via the Ward algorithm using MetaboAnalyst 5.0 (Chong *et al.*, 2018) to identify metabolite clusters with significant accession and accession by developmental differences across development ($p \leq 0.05$). Clusters are identified that display the main 4 trends described in text. -bP = bisphosphate; -P = -phosphate; DOX = deoxyxylulose; Fru = fructose; Gal = galactose; GalN = galactosamine; GalUA = galacturonic acid Glc = glucose; GlcA = glucuronic acid; GlcN = glucosamine; GlcNAc = N-acetylglucosamine; Man = mannose; PEP = phosphoenolpyruvate; PGAs = 2- and 3-phosphoglycerate content, combined; RuBP = ribulose 1,5-bisphosphate. Units are in the generalized log-transformation of pmol mg DW^{-1}.

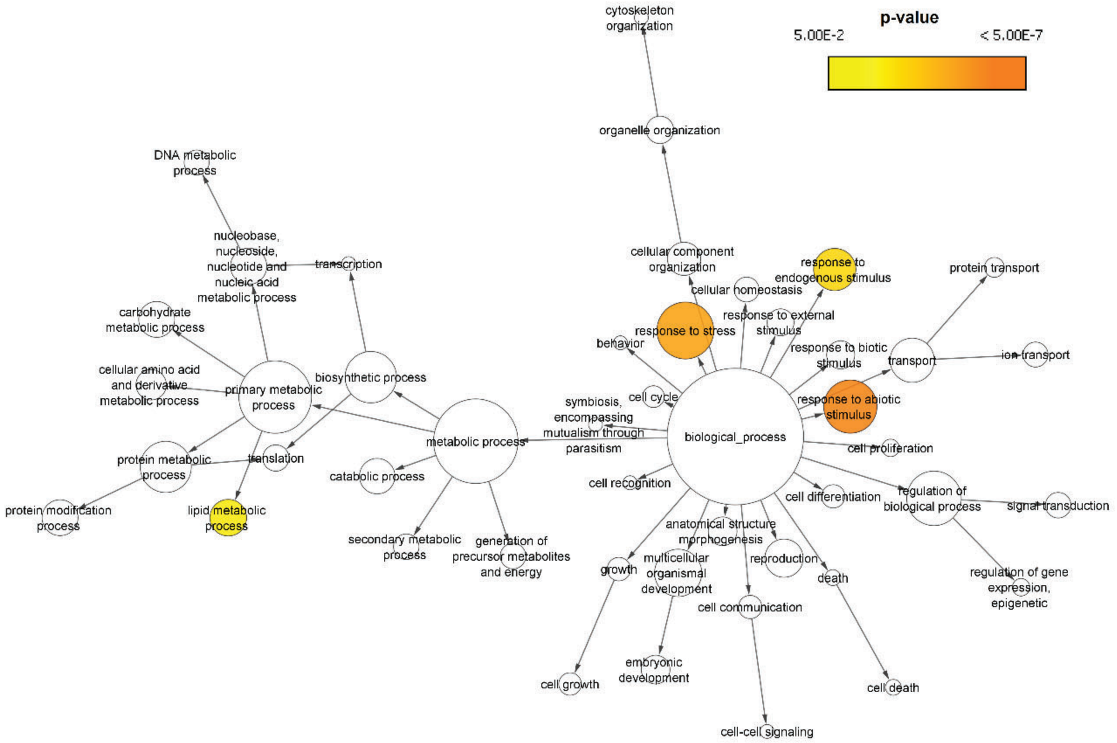

Figure S4. Full profile of gene ontology biological process (GO:BP) results from pool of differentially expressed genes. Filled circles indicate significantly over-represented GO:BP terms via PANTHER GO-Slim, with p-value corresponding to false discovery rate. Functional classification of DEGs based on gene ontology (GO) terms were carried out via PANTHER GO-Slim using the GO biological process (GO:BP) database (Mi and Thomas, 2009; Mi *et al.*, 2021). Visualization of the DEG GO:BP profile was performed using BiNGO (Maere *et al.*, 2005).

Table S1 – gene annotations used throughout manuscript

Hub or hub neighbor genes of unknown domain or function

Ta ID	At ID	Description
Ta1.0_00661	AT5G07170	Cell_cycle_regulated_microtubule_associated_protein_
Ta1.0_01444	AT3G05450	CONTAINS_InterPro_DOMAIN/s:_Cystatin-related,_plant_(InterPro:IPR006525)
Ta1.0_01948	AT5G62400	unknown_protein
Ta1.0_04264	AT5G51250	Galactose_oxidase/kelch_repeat_superfamily_protein_
Ta1.0_04485	AT5G17120	Cystatin/monellin_superfamily_protein_
Ta1.0_04973	AT3G25910	Protein_of_unknown_function_(DUF1644)_
Ta1.0_05161	AT5G13600	Phototropic-responsive_NPH3_family_protein_
Ta1.0_06000	AT3G09380	Protein_of_unknown_function_(DUF59)_
Ta1.0_06202	AT1G72720	Protein_of_unknown_function_(DUF3511)_
Ta1.0_06430	AT2G34580	CONTAINS_InterPro_DOMAIN/s:_Herpesvirus_UL139,_cytomegalovirus_(InterPro:IPR021042)
Ta1.0_07759	AT1G27540	Protein_of_unknown_function_(DUF295)_
Ta1.0_08192	AT1G50950	Thioredoxin_protein_with_domain_of_unknown_function_(DUF1692)_
Ta1.0_08529	AT5G53635	F-box/RNI-like/FBD-like_domains-containing_protein_
Ta1.0_10081	AT4G20095	Protein_of_unknown_function_(DUF626)_
Ta1.0_10356	AT5G57815	Cytochrome_c_oxidase,_subunit_Vib_family_protein_
Ta1.0_12069	AT1G21990	F-box/RNI-like/FBD-like_domains-containing_protein_
Ta1.0_12226	AT5G40190	RNA_ligase/cyclic_nucleotide_phosphodiesterase_family_protein_
Ta1.0_12657	AT2G30000	PHF5-like_protein_
Ta1.0_12661	AT2G30000	PHF5-like_protein_
Ta1.0_12847	AT4G29905	unknown_protein
Ta1.0_13522	AT4G17070	peptidyl-prolyl_cis-trans_isomerases_
Ta1.0_14723	AT4G24330	Protein_of_unknown_function_(DUF1682)_
Ta1.0_15348	AT5G51250	Galactose_oxidase/kelch_repeat_superfamily_protein_
Ta1.0_15692	AT4G19080	Protein_of_unknown_function_(DUF594)_
Ta1.0_17964	AT4G13992	Cysteine/Histidine-rich_C1_domain_family_protein_

Ta1.0_18173	AT1G01550	BPS1_Protein_of_unknown_function_(DUF793)_
Ta1.0_18319	AT1G49750	Leucine-rich_repeat_(LRR)_family_protein_
Ta1.0_18529	AT2G43445	F-box_and_associated_interaction_domains-containing_protein_
Ta1.0_18734	AT5G25450	Cytochrome_bd_ubiquinol_oxidase,_14kDa_subunit_
Ta1.0_18788	AT4G16045	TRAF-like_superfamily_protein_
Ta1.0_18950	AT2G40020	Nucleolar_histone_methyltransferase-related_protein_
Ta1.0_19170	AT1G09650	F-box_and_associated_interaction_domains-containing_protein_
Ta1.0_19505	AT4G28025	unknown_protein
Ta1.0_19918	AT2G04520	Nucleic_acid-binding,_OB-fold-like_protein_
Ta1.0_20282	AT1G58684	Ribosomal_protein_S5_family_protein_
Ta1.0_20894	AT1G45332	Translation_elongation_factor_EFG/EF2_protein_
Ta1.0_21161	AT5G42850	Thioredoxin_superfamily_protein_
Ta1.0_22268	AT5G50450	HCP-like_superfamily_protein_with_MYND-type_zinc_finger_
Ta1.0_23948	AT1G23950	Protein_of_unknown_function_(DUF626)_
Ta1.0_23949	AT1G23950	Protein_of_unknown_function_(DUF626)_
Ta1.0_24246	AT1G20320	Haloacid_dehalogenase-like_hydrolase_(HAD)_superfamily_protein_
Ta1.0_24323	AT2G25420	transducin_family_protein_/_WD-40_repeat_family_protein_
Ta1.0_24433	AT1G64840	Protein_of_unknown_function_(DUF295)_
Ta1.0_25976	AT2G34460	NAD(P)-binding_Rossmann-fold_superfamily_protein_
Ta1.0_26098	AT1G56230	Protein_of_unknown_function_(DUF1399)_
Ta1.0_26343	AT3G32960	Domain_of_unknown_function_(DUF1985)_
Ta1.0_26630	AT3G59910	Ankyrin_repeat_family_protein_
Ta1.0_27085	AT2G27480	Calcium-binding_EF-hand_family_protein_
Ta1.0_27097	AT5G58800	Quinone_reductase_family_protein_
Ta1.0_27105	AT3G23570	alpha/beta-Hydrolases_superfamily_protein_
Ta1.0_27281	AT5G56920	Cystatin/monellin_superfamily_protein_
